# Moving on to older years

**DOI:** 10.1111/aogs.14349

**Published:** 2022-03-30

**Authors:** Reynir Tómas Geirsson

**Affiliations:** ^1^ Professor emeritus, University of Iceland Reykjavik Iceland

As the world's human population is nearing eight billion,^1^ there are definite indications that a slow‐down in population‐growth is likely in this century with rapidly falling birthrates in not only middle‐ and high‐resource countries, but also those with lower resources.^2^ The reasons are largely socioeconomic, related to better survival and longer life expectancies, and also and not least tied to improved education, particularly among women. Outlook on life has shifted, at least in better off societies, and procreation is no longer the same goal in life for everyone.^2^ Environmental impacts affecting hormonal balances and gamete production in both sexes also count in and may do so increasingly in the near future.^2^ After no more than two or three additional billions of people towards the end of this 21st century a balance may hopefully be achieved. This would be good news for the planet if it would at the same time be possible to adopt the vital steps which have been agreed upon by most nations of the world for halting climate changes and global warming, reducing pollution, and providing for greater equity among nations, amidst different groups in society and individuals.^3^ Continuous economic growth in better off societies must also be curtailed. Endless growth will have it's limits, as was pointed out 50 years ago.^4^ Man is the source of most global problems with his insatiable drive for more. Humans have to find the balance by themselves and drastically reduce their own multiplication − not leave the field to viruses that cause pandemics where people perish not only in their thousands, but in millions.

That the SARS Covid virus is a threat to even healthy women in pregnancy is now shown in a landmark nationwide study in this issue of AOGS by Karen Vousden and colleagues from the UK National Perinatal Unit in Oxford, a major epidemiologic center.^5^ It is worthwhile studying this article carefully to note how especially older, overweight and of course unvaccinated mothers do face added risk from Covid infection. Even if the risk is small, it is tangible and measurable. That is no surprise. And if this is the case in a high‐resource country like the UK, then we know that women in poorer societies will have to counter more adversities. In addition to Covid we also now have to live once again with short‐sighted despots who yet again cause untold suffering through war and devastation. When will it end? The world must change course no later than now, we all agree. Yet the universal will to effect that is still far off. While we as individuals have each to add our own contribution, − the rich far more than those who are poorer − in the end systems that govern our habits and daily life must change in order to bring about the wide‐ranging measures required to allow us to survive and progress as humanity.

The falling population growth expected in the next decennia will in addition necessitate widespread adjustment to a relatively high number of older people before the new population equilibrium is reached. Societies, even if rich, will find it problematic to provide for large numbers of older people. New solutions will have to be found to allow the older generations to contribute gainfully and thus sustain themselves, at least in part. Retirement ages between 60 and 70 will not be sustenable and must be raised to higher and flexible levels within the next decade. The majority of people reaching the common retirement age of 67–70 in the Nordic countries are in good health. In other Europan countries retirement ages are even lower. A large majority of these older people feel they have a capacity to carry on, perhaps in less than full employment or in a different scene, while still contributing to society. The former Chief Editor of AOGS writing these words that you are reading, is now well into his seventies, but fortunately in good health. I have enjoyed a more leisurely life and have not missed my former everyday clinical activities, the excitement of the labor ward or that of the operating room. These are now part of the past. I did my share in my time as a clinician and was glad to enter a new phase at the age of almost 68. Being able to continue with some academic activities, to interact with masters‐ and PhD students, to do committee work and contribute to educational matters in medicine has been a blessing. I have as many colleagues of my age developed new interests and activities that may matter a little for others, even with respect to obstetrics and gynecology. Work for some hours most days, including in a Covid‐telephone center giving advice to those stricken by variouly severe viral infection symptoms. Combining more free time with new committments could be a goal for many of us as we grow older, a goal that takes us beyond the golf course or time spent on sunny southern beaches.

Societies have to find ways of making it possible for retirees to carry on after withdrawing from an official and busy position and not make it obligatory to leave. Let us work towards giving older colleagues the chance to use their hard won knowledge and experience gainfully as long as they wish and can.
